# Alcohol Dependence and Genes Encoding α2 and γ1 GABAA Receptor Subunits: Insights from Humans and Mice

**DOI:** 10.35946/arcr.v34.3.10

**Published:** 2012

**Authors:** Cecilia M. Borghese, R. Adron Harris

**Affiliations:** **Cecilia M. Borghese, Ph.D.**, *is a research associate, at the University of Texas at Austin, Austin, Texas.*; **R. Adron Harris, Ph.D.,***is M. June and J. Virgil Waggoner Chair in Molecular Biology and director of the Waggoner Center for Alcohol and Addiction Research, at the University of Texas at Austin, Austin, Texas.*

**Keywords:** Alcohol dependence, alcoholism, genetic factors, DNA, genetic theory of alcohol and other drug use (AODU), genetic vulnerability to AODU, genetic variants, γ-aminobutyric acid (GABA), GABA_A_ receptor (GABA_A_-R) subunits, GABRA2, GABRG1, single nucleotide polymorphisms (SNPs), ion channels, neurotransmitters, gene association studies, human studies, animal studies, mice, rats

## Abstract

One approach to identifying the causes of alcoholism, particularly without crossing ethical boundaries in human subjects, is to look at the person’s genome (and particularly at the variations that naturally arise in the DNA) to identify those variations that seem to be found more commonly in people with the disease. Some of these analyses have focused on the genes that encode subunits of the receptor for the brain chemical (i.e., neurotransmitter) γ-aminobutyric acid (GABA). Different epidemiological genetic studies have provided evidence that variations in certain GABA_A_ receptor (GABA_A_-R) subunits, particularly subunits α2 and γ1, are correlated with alcohol dependence. Manipulations of these genes and their expression in mice and rats also are offering clues as to the role of specific GABA_A_-Rs in the molecular mechanisms underlying alcoholism and suggest possibilities for new therapeutic approaches.

Even though the consequences of alcohol dependence (AD) clearly are devastating and obvious to observers, the molecular mechanisms involved in the development of the disease are far from clear and understood. The search for these mechanisms is made even more difficult by the vast number of genes, proteins, and pathways in the human body that potentially could be involved, and by the obvious limitations of conducting research with human subjects without crossing ethical boundaries. Yet despite these complexities, various approaches already have allowed researchers to gather much knowledge in recent years, and the essential players in alcohol’s mechanisms of action and in the development of AD already may have been identified. Thus, research has found that the primary targets of alcohol seem to be proteins prominently involved in neuronal communication, including:

- Ion channels in the neuronal membrane that are activated by signaling molecules (i.e., neurotransmitters) such as γ-aminobutyric acid (GABA) (i.e., GABA_A_ receptors), glycine (i.e., glycine receptors), glutamate (i.e., *N*-methyl-d-aspartate receptors [NMDA-Rs]), acetylcholine (i.e., nicotinic receptors), and serotonin (i.e., 5-HT_3_ receptors);
- Ion channels regulated by changes in the electric potential across the neuronal membrane (i.e., voltage-gated channels), such as voltage-gated calcium channels; and
- Ion channels regulated by a type of regulatory molecules called G-proteins, such as G-protein–coupled inwardly rectifying potassium channels (GIRKs).

Alcohol’s actions on these primary targets trigger the involvement of other systems that ultimately culminate in the development of dependence (Vengeliene et al. 2008).

Many techniques have yielded insight into alcohol’s effects on the organism, but perhaps the most challenging field, given the logical ethical constrains, is the study of the neuronal structures and mechanisms that are affected by alcohol and/or which play a role in the development of AD in living humans. One way of circumventing these limitations is by studying how the natural variations (i.e., polymorphisms) between individuals in the genomic DNA relate to AD—that is, whether any specific variants are found more or less commonly than would be expected by chance in people with the disorder. This analysis can provide a glimpse of which genes or gene variants contribute to and shape the development of the disorder.

These natural differences in the genomic DNA between individuals arise from spontaneous mutations of single DNA building blocks (i.e., nucleotides) and are called single nucleotide polymorphisms (SNPs). (For more information on SNPs and their analysis, see the sidebar). In the past 10 years, different genetic association studies in alcohol-dependent subjects have identified several genes linked to this condition. Some examples of proteins that are encoded by genes in which the AD-linked SNPs are located include the following:

- The μ-opioid receptor (encoded by the OPRM1 gene) (Bart et al. 2005; Kim et al. 2004; Nishizawa et al. 2006; Ray and Hutchison 2004; Rommelspacher et al. 2001; Zhang et al. 2006);
- The κ-opioid receptor (OPRK1) (Edenberg et al. 2008; Xuei et al. 2006; Zhang et al. 2008);
- Neuropeptide Y (NPY) (Ilveskoski et al. 2001; Lappalainen et al. 2002; Mottagui-Tabar et al. 2005);
- The muscarinic acetylcholine receptor M2 (CHRM2) (Dick et al. 2007; Luo et al. 2005; Wang et al. 2004); and
- The corticotropin-releasing hormone receptor 1 (CRHR1) (Chen et al. 2010).

Another group of genes related to alcohol dependence encode the GABA_A_ receptors (GABA_A_-Rs). This article will summarize what is known about the role of these receptors in the development of alcohol dependence.

## GABA_A_ Receptors

The GABA_A_-Rs are proteins that span the membrane encasing the nerve cells (i.e., neurons) and which are composed of five subunits arranged around a central pore. There are several classes of subunits, including alpha (α), beta (β), gamma (γ), delta (δ), epsilon (ɛ), pi (π), theta (θ), and rho (ρ) subunits.

**Single-Nucleotide Polymorphisms and Their Analysis**

**What Are Single-Nucleotide Polymorphisms?**

Single-nucleotide polymorphisms (SNPs, pronounced “snips”) are spontaneous mutations of single building blocks (i.e., nucleotides) in the genomic DNA. They can occur randomly, in any region of the DNA, including within those regions of the gene that actually encode parts of the resulting protein (i.e., coding sequences), within “silent” regions of a gene that ultimately do not encode parts of the resulting protein (i.e., non-coding regions), or in the regions between genes (i.e., intergenic regions). When a SNP occurs within a coding sequence, it may or may not change the amino acid sequence of the encoded protein. Each amino acid is represented by a three-nucleotide block of DNA (i.e., a codon). Because there are four different nucleotides (represented as A, C, G, and T), 64 possible codons exist; however, these encode only 20 amino acids. As a result, the genetic code is degenerate—that is, several codons may encode the same amino acid (e.g., both ACT and ACC encode threonine). A SNP in which both the original codon and the mutant codon produce the same protein sequence is called a synonymous polymorphism or silent mutation. If a different polypeptide sequence is produced, it is called a replacement polymorphism. This can result either in the introduction of a different amino acid, which is called a missense mutation, or in a premature stop of the protein, which is called a nonsense mutation (see the figure). Even if the SNP occurs in a noncoding region of the gene, it still may affect regulatory processes that could result, for instance, in altered protein levels.

When aligning DNA sequences from different individuals and comparing them at the same positions (i.e., loci) in the DNA, the occurrence of a SNP results in different “versions” of DNA called alleles. For example, in the figure, the two alleles for the SNP rs279868 are “A” and “G”. Alleles frequently are transmitted from one generation to the next in a larger DNA block, usually from 5,000 to 100,000 nucleotides long. These blocks, which can contain numerous SNPs, are known as haplotypes. Thus, a haplotype specifies markers on one member of a pair of homologous chromosomes (i.e., either the chromosome inherited from the mother or the one inherited from the father).

Haplotypes are not always transmitted from one generation to the next, however, because of a process called recombination that randomly occurs during the formation of germ cells. As a result of recombination, new haplotypes should be formed based on the frequencies of the different alleles involved in the general population. Sometimes, however, certain combinations of alleles occur more or less frequently in a given population than would be expected from random formation of haplotypes. This nonrandom association of alleles at two or more loci is referred to as linkage disequilibrium. Identification of alleles that are in linkage disequilibrium can be useful for determining genes that are involved in conditions such as alcohol dependence.

**How Can SNPs Be Analyzed?**

Some genetic studies try to determine whether a certain allele (or haplotype) is present more or less frequently in people who suffer from a medical condition (e.g., alcoholism) than those without the disease; these are called genetic-association studies. One type of genetic-association studies are case–control studies, which include both individuals affected by the condition and disease-free control individuals from the same population. The frequency of alleles then is determined in both case and control subjects. Differences in the frequency of an allele between the two groups suggest that an association exists between the involved gene and the medical condition, with a certain allele conferring an increased or decreased risk for the condition. Identification of the precise nature of this association then requires additional studies.

For example, the allele may alter the sequence, the splicing, or the levels of expression of the protein encoded or it may be in linkage disequilibrium with another allele that constitutes the genetic basis for the difference.

A caveat of these studies is that the frequencies of alleles/haplotypes can vary with ethnicity and geography; this is known as population stratification. One way to avoid this problem is to use family-based association designs. In this situation, unaffected family members (e.g., parents or siblings) are used as control subjects for the affected individuals. If an allele increases the risk of having the disease, then that allele would appear more frequently in the affected family members than in the unaffected members.

Other studies, such as twin and adoption studies, focus on the interaction between genes and environment. Twin studies compare the similarity of identical (i.e., monozygotic) and fraternal (i.e., dizygotic) twins. Identical twins generally are more similar than fraternal twins, because they are not only exposed to the same environment but also share a higher genetic similarity. In adoption studies, the adopted individuals are compared with control individuals (i.e., non-adopted individuals either from the adoptive family or the general population or adopted but unrelated children in the adoptive family). By comparing large numbers of twin pairs or adoptees and control subjects (e.g., with respect to the frequency of certain SNPs as well as the disease of interest), it is possible to better understand the role of genes and environment in the characteristics of a person.

Another approach to using SNPs to identify genes involved in a certain disease is to conduct genome-wide association studies (GWASs). With this strategy, a genetic association with the disease is investigated using many SNPs that cover the entire genome, instead of just a few genes as in the study approaches described above. This allows researchers to identify associations with genes that previously had not been expected to play a role in the disease under investigation. The downside of the GWASs is that they require a much larger number of subjects than do the other studies.

**Figure:**
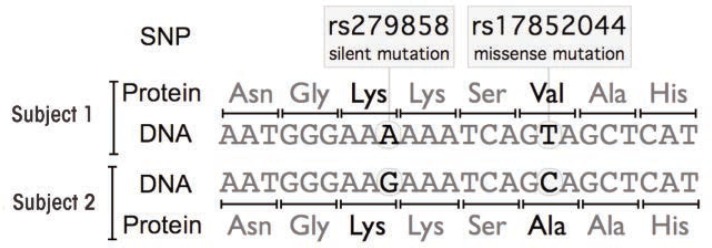
Segment of a single strand of DNA representing a fragment of the coding region from the *GABRA2* gene from two different people. There are two SNPs in this gene region—one in which both variants of the DNA encode the same amino acid (i.e., a silent mutation) and one in which the two variants of the DNA encode different amino acids (i.e., a missense mutation).

The most commonly found GABA_A_-Rs consist of two α, two β, and one γ or δ subunit. For some classes of subunits, several variants exist that are encoded by different genes, including six for the α subunit, three for the β subunit, and three for the γ subunit, allowing for numerous different subunit combinations. When GABA binds to the GABA_A_-R, it activates the receptor and the central channel opens, allowing the entrance of negatively charged ions (i.e., anions), specifically Cl^−^, to enter the neuron. This results in an increase in the difference in electrical charge between the inside and outside of the neuron (i.e., hyperpolarization), which in turn makes it more difficult for the neuron to transmit a nerve impulse, thus ultimately leading to inhibition of neuronal activity. Accordingly, GABA is considered an inhibitory neurotransmitter.

Considerable evidence points to the GABA_A_-R as one of the main targets of alcohol (Kumar et al. 2009). The most abundant subunit combination in the brain, α1β2γ2, has been the most studied. Recently, the δ subunit–containing GABA_A_-Rs also have been scrutinized in relationship to alcohol (Lobo and Harris 2008).

The genes encoding the GABA_A_-R subunits are located in clusters on different chromosomes, including one cluster on chromosome 4 that carries genes called *GABRB1*, *GABRA4*, *GABRA2*, and *GABRG1*, which encode the β1, α4, α2, and γ1 subunits, respectively (see the figure). Previous human genetic studies have linked genetic polymorphisms in two regions of the *GABRA2* gene (i.e., in the middle and at the 3′ end of the gene) and in the region between the *GABRA2* and *GABRG1* genes (i.e., the *GABRA2* to *GABRG1* intergenic region) with AD (Agrawal et al. 2006; Covault et al. 2004; Edenberg et al. 2004; Enoch et al. 2006; Fehr et al. 2006; Lappalainen et al. 2005; Soyka et al. 2008). However, the evidence is not unequivocal, because other studies found no association between AD and the SNPs in this area (Covault et al. 2008; Drgon et al. 2006; Matthews et al. 2007). Even among the studies that did find a correlation, some inconsistencies existed. For instance, the first study of the association between *GABRA2* and AD came from the Collaborative Study on the Genetics of Alcoholism (COGA), a vast family-based association study (Edenberg et al. 2004). When Agrawal and colleagues (2006) extended the study of the COGA sample to include illicit drug dependence and comorbid dependence on alcohol and other drugs, the association was found only in subjects with AD and co-occurring drug dependence. On the other hand, Covault and colleagues (2004) found that the association was stronger when alcoholics with comorbid drug dependence were removed from the sample. Despite these inconsistencies, however, most of the clinical and genetic evidence points to *GABRA2* as a major genetic player in AD (see Enoch 2008).

All the SNPs that have been studied in the *GABRA2* and *GABRG1* genes to date are nonfunctional polymorphisms—that is, they do not alter the amino acid sequence of the encoded proteins. An alternative explanation for their role in the development of AD would be that the SNPs may alter the amount of protein that is produced. To address this possibility, researchers have analyzed the levels of an intermediate molecule called messenger RNA (mRNA) that is generated when the information encoded in the DNA is used for the production of a functional protein (i.e., during gene expression). An analysis of α2 mRNA levels in the prefrontal cortex of AD and control subjects found an association between α2 mRNA levels and the different variants (i.e., alleles) in the SNP rs279858,^1^ although mRNA levels did not differ between control subjects and alcoholics (Haughey et al. 2008). Other recent data suggest that the apparent correlation between AD and *GABRA2* may result from a linkage disequilibrium with a not-yet-detected functional variant in the neighboring *GABRG1* gene. These findings on *GABRA2* and *GABRG1* and their association with AD are reviewed in the following sections, focusing on human studies and correlates in genetically modified rodents.

## Analyses of the *GABRA2* and *GABRG1* Genes

### Genetic-Association Studies

Several gene-association studies have examined the relationship of the *GABRA2* and *GABRG1* genes with AD, with varying results. Two studies—a large twin sample of the Australian population that also investigated the association with smoking and illicit drug use (Lind et al. 2008), and a small case–control study in an Italian sample (Onori et al. 2010)—reported no association between *GABRA2* and AD. Another case–control and family-association study (Sakai et al. 2010) sought to analyze the correlation between *GABRA2* genotype and substance abuse and behavioral problems in adolescents. The investigators only analyzed a single SNP in *GABRA2*, which was found not to be associated with conduct disorder or AD in adolescents.

Enoch and colleagues (2010) conducted a case–control study in African-American men with single and comorbid diagnoses of alcohol, cocaine, and heroin dependence, assessing the *GABRA2* genotype as well as childhood trauma. The exposure to childhood trauma predicted substance dependence. common in control subjects and seemed to confer resilience to addiction after exposure to severe childhood trauma. These findings suggest that in African-American men, childhood trauma, *GABRA2* SNPs, and their interaction determine (at least in part) the risk of or resilience to substance dependence. However, the data did not show a direct association between *GABRA2* and AD.

In a case–control adoption study of substance abuse (Philibert et al. 2009), the researchers determined the participants’ genotypes for SNPs encompassing the *GABRA2* gene and analyzed them with respect to their history of alcohol, nicotine, and/or cannabis dependence. Both *GABRA2* genotype and haplotype were significantly related to vulnerability to all three types of substance dependence, particularly nicotine, and this association was more pronounced in female than in male subjects.

A small study using Japanese subjects (mostly social drinkers) examined the association between genetic variation in *GABRA2* (as assessed via seven SNPs) and subjective responses to alcohol as well as stimulant and sedative effects of alcohol (Roh et al. 2010). Three of these seven SNPs, all of which were located in the middle of the *GABRA2* gene, showed significant associations with subjective effects of alcohol. Specifically, individuals carrying one or two copies of the more common *GABRA2* allele (which is not associated with AD) showed greater subjective responses to alcohol than did individuals carrying two copies of the allele associated with AD. These results are, to some extent, in agreement with previous studies (Haughey et al. 2008; Pierucci-Lagha et al. 2005).

Another study (Kareken et al. 2010) examined the association between *GABRA2* SNPs and the brain’s reward system. The participants, which included social drinkers, heavy drinkers, and alcohol-dependent individuals, first were assessed with respect to the brain’s responses to alcohol cues (i.e., exposure to the odor of their preferred alcoholic beverage or a control odor) under both alcohol intoxication and control conditions using an imaging technique called functional MRI (fMRI). Then, the subjects were stratified according to their genotype at a SNP in *GABRA2* that previously had been shown to be associated with AD (Edenberg et al. 2004). All participants carried at least one copy of the high-risk allele of the SNP. Under both alcohol intoxication and control conditions, participants with two copies of this allele (i.e., homozygous subjects) exhibited a larger response to alcoholic odors than to control odors in one brain region (i.e., the medial frontal cortical areas), whereas participants with only one copy of this allele (i.e., heterozygous subjects) exhibited a larger response in another brain area (i.e., the ventral tegmental area). Thus, *GABRA2* variants seem to modify the activation of reward-related areas after exposure to alcohol-associated cues. Another study (Villafuerte et al. 2011) used fMRI to analyze the relationship between two *GABRA2* SNPs, the personality trait of impulsivity, and activation of a brain region called the insula cortex during anticipation of reward or loss in a family sample with high numbers of alcohol-dependent individuals. The investigators detected an association of all three variables, suggesting that *GABRA2* genotype influences insula responses and therefore impulsivity.

Another type of study called linkage disequilibrium analyzes whether certain alleles located close to each other on the same chromosome are inherited together more or less frequently than would be expected by chance alone.^3^ Such studies in different populations have focused on *GABRA2* and either *GABRG1* (Ittiwut et al. 2008) or the intergenic region between the two genes (Philibert et al. 2009). The findings of these studies led to the conclusion that associations observed between *GABRA2* and the condition under investigation could be attributable to functional genetic variation at the *GABRG1* locus or that disease-related variants may exist at both loci.

Some new studies have focused on the *GABRG1* gene. Ray and Hutchinson (2009) examined associations between two SNPs of the *GABRG1* gene and alcohol use in hazardous drinkers. The data indicated that variation in one of the SNPs was associated with level of response to alcohol, drinking behavior, and alcohol problems.

Additional evidence of a significant *GABRG1* association with AD was found in a study involving Finnish Caucasian and Plains American Indians that examined both the *GABRA2* and *GABRG1* genes (Enoch et al. 2009). In both populations, there were significant haplotype and SNP associations of *GABRG1,* but not *GABRA2,* with AD. However, in the Finnish study population, the association of three less common haplotypes with AD was determined by *GABRA2*. Taken together, the findings of all of these studies suggest that independent contributions from both *GABRG1* and *GABRA2* likely contribute to the risk of AD.

### Genome-Wide Association Studies

In contrast to the approaches used in the studies described above, genome-wide association studies (GWASs) investigate the genetic association with a disease using many SNPs that cover the entire genome instead of just a few genes. GWASs therefore also may discover associations with genes not previously suspected to be involved in the disease. One GWASs (Bierut et al. 2010) identified 15 SNPs that showed a significant association with AD. Moreover, when the investigators performed an independent evaluation for *GABRA2*, they found that five SNPs at that gene showed a modest association with AD.

Another GWASs was carried out in a case–control sample drawn from the families in the COGA, using individuals with AD (56 percent of whom also were dependent on illicit drugs) and individuals who used alcohol but were not dependent on alcohol or illicit drugs (Edenberg et al. 2010). The study identified no single SNP that met genome-wide criteria for significance; however, several clusters of SNPs provided mutual support for an association with the disease. An analysis of SNPs in genes encoding GABA_A_-R subunits in this sample found that a SNP in a gene called *GABRR2,* which encodes the GABA_A_-R α2 subunit, was highly correlated with AD in this GWASs. This supported previous results, even though the level of significance was not high enough for genome-wide significance (Xuei et al. 2009). Likewise, SNPs in *GABRG1* were associated with AD, consistent with previous studies (Covault et al. 2008; Enoch et al. 2009). However, there was no evidence that the neighboring *GABRA2* gene was associated with AD. Finally, SNPs in other genes encoding GABA_A_-R subunits (i.e., *GABRG3,* which encodes the γ3 subunit; *GABRA1,* which encodes the α1 subunit; and *GABRG2,* which encodes the γ2 subunit) also were associated with AD, again confirming findings of other investigators (Dick et al. 2004, 2006).

It is important to note that there are some inconsistencies in the findings of these genetic studies, which can be attributed to the inherent differences among the study types, the variability (i.e., heterogeneity) of the disease, and the genetic differences among the populations studied. In general, however, evidence continues to accumulate supporting an association between variations in genes encoding GABA_A_-R subunits, particularly α2 and γ1 subunits, and AD.

### Studies in Genetically Engineered Rodents

The studies in humans discussed above have been supplemented with studies of alcohol’s effects on behavior in genetically engineered mice carrying mutations in GABA-related genes (Crabbe et al. 2006). For example, Boehm and colleagues (2004) found that mice in which the GABA_A_-R α2 subunit had been deleted (i.e., α2 knockout mice) differed from control mice both in behavioral tests conducted without alcohol exposure (e.g., showed decreased spontaneous locomotion when tested for locomotor response to novelty) and in some behavioral responses to alcohol. Thus, the α2 knockout mice showed a shorter duration of alcohol-induced loss of righting reflex (LORR), which is a measure of alcohol’s hypnotic effects. However, the sensitivity of the mice to acute ethanol withdrawal seemed unchanged, as did alcohol’s anxiety-reducing (i.e., anxiolytic) effects. This lack of an effect of α2 deletion on alcohol’s anxiolytic effects was unexpected, because mice genetically modified to possess a benzodiazepine-insensitive α2 subunit called α2(H101R) (i.e., knockin mice) no longer exhibited anxiolytic behavior when they were treated with the benzodiazepine diazepam (Low et al. 2000). Both alcohol and benzodiazepines are anxiolytic, and both increase GABA_A_-R function. If both benzodiazepines and alcohol acted through α2-containing GABA_A_-Rs to produce anxiolytic effects, deletion of the α2 subunit in the knockout mice should reduce alcohol’s anxiolytic effects, and that did not happen. Furthermore, accurate assessment of alcohol’s anxiolytic effects in the α2 knockout mice was complicated by the altered locomotor responses in these animals. Finally, female α2 knockout mice, but not males, preferred and consumed less alcohol than did the controls. However, interpretation of these findings is complicated by the female mice’s greater aversion to bitter-tasting substances.

Another study was conducted in knockin mice carrying α1, α2, α3, or α5 GABA_A_-R subunits that are insensitive to benzodiazepines through a mutation in a single amino acid (Tauber et al. 2003). The investigators administered diazepam and alcohol in combination to these mice and then determined the mice’s LORR. All animals except for the α2 (H101R) mice showed similar sensitivity (i.e., increased LORR and reduced locomotor activity) to the combined drugs. Furthermore, the α2 (H101R) mice exhibited normal responses to alcohol alone (i.e., normal LORR and locomotor activity) and to a combination of low-dose alcohol and diazepam (i.e., normal locomotor activity). Thus, the benzodiazepine-induced increase in the alcohol-mediated hypnosis depends on the α2 GABA_A_-R subunit.

Although null mutant mice that completely lack a certain GABA_A_-R subunit provide important contributions to our knowledge of alcohol’s targets, the deletion of a receptor in the brain, particularly an important one, is likely to trigger compensatory changes. For example, other receptor subunits could become more abundant and take on the functions of the missing subunit. An alternative approach is to design specific mutations that will render that receptor insensitive to alcohol but normal in every other aspect. One study compared the activities of GABA_A_-Rs containing wild-type and mutated α2 subunits expressed in frog egg cells (i.e., *Xenopus* oocytes) (Blednov et al. 2011). With the normal GABA_A_-Rs containing the wild-type subunit α2 (SL),^4^ submaximal GABA responses were enhanced (i.e., potentiated) by alcohol. However, this potentiation was absent in the mutant α2 (HA)-containing GABA_A_-Rs; there even was a small inhibition of the receptor’s activity. In contrast, the mutation did not affect the receptor’s sensitivity to GABA or the modulation by zinc, the benzodiazepine flunitrazepam, or the anesthetic etomidate (Werner et al. 2011).

On the basis of these findings, researchers developed and studied two corresponding mouse lines, the α2 SL/SL (i.e., wild-type) mice and the α2 HA/HA (i.e., knockin) mice. The responses to alcohol in these animals then were studied using a variety of tests (Blednov et al. 2011). The analyses found that some typical effects of alcohol (e.g., conditioned taste aversion and motor stimulation) were absent in the knockin mice. Moreover, the knockin animals showed changes in alcohol intake and preference in multiple tests as well as increased alcohol-induced hypnosis. In contrast, the knockin animals exhibited no changes in alcohol’s anxiolytic and motor incoordination effects. These altered behavioral responses to alcohol in mutant (i.e., both knockout and knockin) mice may be related to altered subjective effects of alcohol in humans with different α2-associated SNPs (Kareken et al. 2010; Roh et al. 2010). In summary, the study suggests that α2-containing GABA_A_-Rs may be responsible for specific alcohol-induced effects. A subsequent study of the changes in mRNA levels induced by these mutations in the α2 subunit in the outer layer of the brain (i.e., the cerebral cortex) underlines the advantages of using knockin over knockout mice. Of almost 11,000 probes tested, the expression of only three genes was significantly modified in the knockin mice, and the behavioral responses to the sedative agents pentobarbital and flurazepam were unchanged (Harris et al. 2011). This confirms that the introduction of these mutations has minimal impact on the knockin animals compared with controls, minimizing the risk that effects unrelated to the behavior being investigated confound the results.

Another study in mice focused on the role of the GABA_A_-R α2 subunit in changes in behavior produced by adaptation to chronic cocaine’s effects (i.e., cocaine behavioral plasticity), such as locomotor sensitization, as well as in addiction (Dixon et al. 2010). In GABA_A_-R α2 null mutant mice, cocaine did not induce a greater effect after repeated administration (i.e., did not produce behavioral sensitization) as it did in wild-type mice. Conversely, in mice carrying the benzodiazepine-insensitive GABA_A_-R α2 (H101R) subunit, an agent called Ro 15–4513 that can increase the receptor responses in this mutant α2 subunit could stimulate locomotor activity if it was delivered into a brain region called the nucleus accumbens and induced behavioral sensitization to this effect after repeated administration. These results suggest that activation of α2-containing GABA_A_-Rs in the nucleus accumbens is sufficient and necessary for behavioral sensitization. Furthermore, the investigators conducted a genetic case–control study in a diverse population (mainly Caucasian) that demonstrated an association of *GABRA2* with cocaine addiction in humans, emphasizing the relevance of α2-containing GABA_A_-Rs in drug dependence.

Finally, researchers used an established animal model of human alcohol abuse, the selectively bred alcohol-preferring (P) rats, to look at the role of GABA_A_-R subunits in alcohol’s effects (Liu et al. 2011). The levels of GABA_A_-R α1 subunits are elevated in a brain region called the ventral pallidum of these rats, and both α1 and α2 levels are increased in another region called the central nucleus of the amygdala (CeA). The study used molecules known as small-interfering RNAs (siRNA), which can interfere with gene expression, to specifically prevent production of α1 and α2 subunits. When siRNA targeted to α2 was infused into the CeA of P rats, both α2 expression and GABA_A_-R density were reduced, and this was associated with inhibition of binge drinking. In contrast, siRNA targeted to α1 did not cause any of these changes when introduced in the CeA but did reduce α1 expression and binge drinking when administered into the ventral pallidum. These results highlight that not only the kind of GABA_A_-R subunit but also the brain region in which it is located are relevant for alcohol consumption.

## Implications of Genetic Findings for Therapeutic Approaches

Extending a previous study (Bauer et al. 2007), Das and colleagues (2010) analyzed the association between a SNP in the *GABRA2* gene and the efficacy of three psychotherapies for alcoholism (i.e., motivational enhancement therapy, cognitive–behavioral therapy, or 12-step facilitation) in preventing extreme drinking in AD patients. The study found that men with a high-risk *GABRA2* allele had a significantly higher probability of extreme drinking than did men without that allele. However, both men and women carrying at least one high-risk allele responded better to the therapy than did those who were homozygous for the low-risk allele. Among the female participants, the most effective therapy was cognitive–behavioral therapy, whereas among male subjects motivational enhancement therapy was most effective.

Tailoring pharmacotherapy to alcohol-dependent patients on the basis of genetic indicators also may be within reach. Two medications, naltrexone and acamprosate, currently are used for the treatment of alcoholism, but often with limited success. In an effort to identify potential associations between genotype and treatment outcome, Ooteman and colleagues (2009) determined SNPs in genes encoding different receptors involved in AD processes (i.e., opioid, dopamine, glutamate, and GABA_A_ receptors) in alcohol-dependent individuals randomly assigned to acamprosate or naltrexone treatment. The investigators also quantified treatment effectiveness using tests administered the day before treatment initiation and on the last day the medication was administered. The tests included a cue exposure (i.e., participants were exposed to the sight and smell of their favorite alcoholic beverage while listening to a mood-induction script), followed by an assessment of self-reported cue-induced craving and physiological cue reactivity (i.e., heart rate). Significant association effects were found for several SNPs, suggesting that this may be the first step in matching patients to pharmacotherapy based on GABA_A_-Rs and other genotypic markers.

## Summary

Studies of genetically modified mice and rats have demonstrated that manipulation of α2 GABA_A_-R subunits produces changes in alcohol-related phenotypes. The findings were more equivocal in human genetic studies, although strong evidence suggests that several GABA_A_-R subunits, particularly α2 and α1, have a role in AD in humans. Future studies hopefully will elucidate what exact mechanism creates these variations in the genetic code that affect AD, and how such variations can be used to provide a path to individualized therapy for patients with AD.

## Figures and Tables

**Figure:**
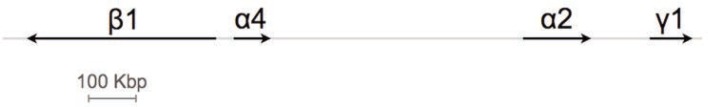
Schematic representation of the cluster of GABA_A_ receptor genes on chromosome 4. Arrows indicate gene position, size, and direction of transcription. The subunits and the names of the corresponding genes are α2 *(GABRA2),* α4 *(GABRA4),* β1 *(GABRB1),* and γ1 *(GABRG1)*.

## Footnotes

1 SNP rs279858 encodes a silent mutation (i.e., a mutation that does not result in an altered amino acid sequence) in the coding region of GABRA2.

3 Even genes located close to each other on a chromosome may not always be inherited together, because of a process called genetic recombination that occurs with a certain probability during the generation of the germ cells.

4 The wild-type α2 (SL) subunit carries the amino acid serine at position 270 and the amino acid leucine at position 277. In contrast, the mutant α2 (HA) subunit carried the amino acids histidine at position 270 and alanine at position 277.
